# Efficacy of light therapy for perinatal depression: a review

**DOI:** 10.1186/1880-6805-31-15

**Published:** 2012-06-06

**Authors:** Shannon K Crowley, Shawn D Youngstedt

**Affiliations:** 1 Department of Exercise Science, Arnold School of Public Health, University of South Carolina, Columbia, SC, USA; 2 WJB Dorn VA Medical Center, Columbia, SC, USA; 3 Department of Psychology, University of South Carolina, Columbia, SC, USA

**Keywords:** Perinatal depression, Postpartum depression, Antepartum depression, Light therapy, Light

## Abstract

Perinatal depression is an important public health problem affecting 10% to 20% of childbearing women. Perinatal depression is associated with significant morbidity, and has enormous consequences for the wellbeing of the mother and child. During the perinatal period, treatment of depression, which could affect the mother and child during pregnancy and lactation, poses a complex problem for both mother and clinician. Bright light therapy may be an attractive treatment for perinatal depression because it is low cost, home-based, and has a much lower side effect profile than pharmacotherapy. The antidepressant effects of bright light are well established, and there are several rationales for expecting that bright light might also be efficacious for perinatal depression. This review describes these rationales, summarizes the available evidence on the efficacy of bright light therapy for perinatal depression, and discusses future directions for investigation of bright light therapy as a treatment for perinatal depression.

## Prevalence and treatment of perinatal depression

Perinatal depression refers to depressive syndromes with onset during pregnancy or within 12 months after delivery [1]. Research findings suggest that between 10% and 20% of pregnant women experience a new episode of depression during pregnancy (antepartum depression) [2-4].

The prevalence of postpartum depression (PPD) is also estimated to be between 10% and 20% [3,5-7], with higher rates among women who had suffered from antepartum depression. Rates of perinatal depression seem to be particularly high in women with a prior history of depression, with estimates ranging from 25% to 50% [8]. Additional psychosocial factors which may increase a woman’s risk for development of perinatal depression include single marital status, chronic illness, alcohol use during pregnancy, and lower socioeconomic status [9].

As during other times, risk of depression during the perinatal period may be influenced by genetic vulnerability. Additionally, the perinatal period may be a time of substantial vulnerability to affective illness due to the rapid fluctuation in hormone levels during pregnancy, and more dramatically, during the postpartum period [9,10]. During pregnancy, depression and co-morbidities such as stress and anxiety appear to have a direct adverse biological impact on the intrauterine environment, and are linked to obstetric complications such as preterm labor, pre-eclampsia, and anemia, as well as adverse neonatal outcomes such as low birth weight, neonatal distress, and other neonatal abnormalities [11-13]. Long-term child health outcomes affected by antepartum depression include increased risk for neurodevelopmental disorders, motor difficulties, emotional and behavioral problems, and adolescent depression [11,12,14].

Postnatally, maternal parenting behavior may be adversely affected by PPD, and this can have detrimental consequences on child behavioral development. Children of mothers who had suffered PPD are at increased risk for development of depression, anxiety disorders (including panic disorder), emotional maladjustment patterns, violent behavior, and other conduct disorders such as attention deficit hyperactivity disorder [15-18].

Treatment of depression during pregnancy and postpartum poses a complex problem for both mother and clinician. Many mothers feel guilty or fearful of their feelings during a time in which they assume they should be happy, thus refusing to acknowledge depressive symptoms and opting to ‘suffer in silence’. Antidepressant use during pregnancy has been linked to multiple harmful effects to the infant, including persistent pulmonary hypertension, cardiac abnormalities, and neonatal withdrawal and toxicity [19-21].

Antidepressant use in mothers could also potentially harm the breastfeeding infants. A recent meta-analysis of 67 studies of antidepressant levels in breastfeeding infants found that out of 15 different antidepressants, all were detectable in breast milk at varying levels [22]. However, the available evidence on the effects of antidepressants during lactation on drug levels in the nursing infant is limited, and largely derived from short duration studies with small sample sizes. Studies have shown that antidepressant drugs or their metabolites are not always found in the child’s serum, even though they may be detectable in breast milk [22,23]. For example, studies have found that levels of sertraline in infant serum were low to negligible in breastfeeding mother-infant pairs, in which the mothers were taking clinically relevant doses of sertraline [24,25], one of the most commonly prescribed antidepressants for breastfeeding mothers. However, it is unknown whether nursing infants might be sensitive to these low levels, particularly with chronic exposure. To date, there has been virtually no investigation of long-term child health outcomes from exposure to maternal antidepressant medication during breastfeeding. This is especially true for the modern selective serotonin reuptake inhibitors (SSRIs), as there are no studies with adequate follow-up of long-term child health outcomes [23,26]. It is noteworthy that no medication has been FDA-approved for PPD [11].

Notwithstanding the evidence of risks of antidepressant treatment for perinatal depression, many mental health providers have concluded that the detrimental effects of untreated maternal depression might pose an even greater threat to maternal and child health outcomes. There is some thought that adverse effects of antidepressant treatment on the nursing infant can be minimized by scaling back the dose [4], though this strategy might also reduce the efficacy of the drug for the mother.

Nonetheless, legitimate concerns about possible adverse effects of antidepressant medications on the developing fetus or newborn have led many women to refuse pharmacotherapy during pregnancy or while breastfeeding. For mothers who choose to take antidepressants during the postpartum period, many forgo the myriad benefits of breastfeeding in order to take antidepressants.

Psychotherapy is apparently an effective treatment for perinatal depression [27,28]. However, due to logistical issues, such as childcare arrangements and expenses, women with perinatal depression might also be less suitable for psychotherapy than other depressed individuals. Estimates indicate that only about 15% of women with PPD are receiving treatment [29]. Thus, alternative or adjuvant treatments for perinatal depression are needed.

## Rationale for bright light therapy of perinatal depression

Bright light therapy may be an attractive treatment for perinatal depression because it is low cost, home-based, and has a much lower side effect profile than pharmacotherapy [30]. Moreover, the efficacy of bright light for other types of depression has been well established.

Particularly well-documented is the efficacy of bright light therapy for winter depression, for which bright light is generally regarded as the first line of treatment [31]. The utility of light for winter depression also has considerable intuitive appeal, as seasonal variation in mood are experienced my much of the population, and clearly attributed to fluctuations in light [32].

Less well recognized is that, in industrial societies, average levels of exposure to bright light (> 1,000 lux) average only about 1 h per day even in good weather conditions [33], and these levels might be inadequate for mood regulation in susceptible individuals. Moreover, accumulating evidence indicates that bright light might be equally efficacious for non-seasonal depression [30,34,35], as well as other morbidities, including disturbed sleep [36,37], fatigue [38,39], and neuroendocrine abnormalities [40].

There are several factors related to the pathophysiology of depression and response to light which might make bright light especially suitable for perinatal depression. First, women with perinatal depression might receive very low light exposure. Pregnancy, particularly in late gestation, may make mobility difficult for some women, thus reducing time spent outdoors. After delivery, women may also experience lower levels of illumination, with increased time spent indoors caring for their infant who may often sleep during the day. Consistent with this notion are findings of seasonality of PPD, with increased risk of PPD associated with delivery during fall and early winter months [41,42] when daylight is of significantly shorter duration. Although a study by Wang *et al*. did not confirm differences in light exposure of postpartum women *vs.* matched control women who were not within 1 year postpartum [43], this remains a tenable hypothesis.

Second, there is some evidence that circadian malsynchronization is associated with depression and resynchronization is associated with the antidepressant effects of bright light [44]. There are reasons to suspect that women with perinatal depression might have circadian malsychronization. During pregnancy and postpartum, new mothers may spend a greater part of the day in dim light conditions in an effort to get sleep they may not be getting during the night. These conditions may be even more exaggerated in perinatal depression, where fatigue and anhedonia may severely limit daily activities. There has been limited investigation of the chronobiology of perinatal depression, though one recent study suggests that both amplitude and timing of melatonin secretion may be dysregulated in women with perinatal depression compared to non-depressed perinatal women [45].

Third, there is emerging evidence that impaired activation of serotonergically targeted circuits may be involved in the pathophysiology of PPD [46,47], which could be corrected with bright light treatment. For example, there is recent fMRI evidence that activation in these areas is abnormal in depressed *vs.* non-depressed mothers when responding to their newborns’ cries [48,49]. Indeed, previous studies have implicated alterations in tryptophan (a serotonin precursor), estrogen, and hypothalamic pituitary adrenal (HPA) axis activity during the perinatal period as possible mediators of serotonin dysregulation [50-53]. Additionally, studies have found that genetic polymorphisms of the 5-HTT gene are associated with variations in PPD [54,55], and increased vulnerability to development of depression in pregnancy and the postpartum [9]. Conversely, the SSRIs have been prescribed for perinatal depression [7], and have resulted in improvements in maternal role functioning in women with PPD [56,57].

There is some compelling evidence that the antidepressant effect of bright light therapy is mediated by serotonergic mechanisms. Studies have shown that the antidepressant effects of light therapy are reversed following tryptophan depletion [58]. Because tryptophan is a precursor to serotonin synthesis, tryptophan depletion reduces availability of serotonin in the brain, which in turn has been associated with emergence of depressive symptoms [59]. Using this established paradigm, recent work has illustrated that the mood lowering effect of tryptophan depletion in healthy women is completely blocked by carrying out the study in bright light (3,000 lux) *vs.* dim light (10 lux) [60]. Bright light therapy may provide a means to improve regulation of the serotonergic system in women with perinatal depression, and bright light therapy poses far less risk to the developing baby and newborn than current pharmacotherapy.

Fourth, there is evidence that perinatal depression may be influenced by alterations in estrogen, which could be corrected with bright light. The close relationship between estrogen and the serotonin system may provide some rationale for findings that some women have increased susceptibility to mood symptoms during periods of hormonal fluctuation, and that estrogen treatment has been shown to be effective in mood disorders, including postpartum depression [61,62]. It is clear from previous work that sex hormones such as estrogen and progesterone - which rise greatly during pregnancy, and then show a sharp drop immediately after parturition - may play a role in the etiology of perinatal depression [9].

There is limited evidence that bright light therapy may be associated with stimulation of luteinizing hormone (LH), a gonadotropin involved in the production of estrogen [63]. The therapeutic benefit of light therapy for perinatal depression may therefore involve modulation of estrogen via stimulation of LH.

Fifth, bright light could alleviate co-morbidities of fatigue and sleep disturbance which might be especially problematic for perinatal depression, when the effects of sleep loss have consequences for both mother and baby. There appears to be an association between short sleep duration and adverse maternal and fetal outcomes [64-66], and limited evidence also suggests an association between sleep problems and maternal depression [67]. Indeed, fatigue and sleep disturbances are used diagnostically in the evaluation of perinatal depression, and are some of the most commonly reported symptoms in women with PPD [68]. Studies indicate that women with perinatal depression report substantially poorer sleep than matched healthy antepartum and postpartum women [69-72]. Evidence indicates that bright light therapy may decrease daytime fatigue partly via improvement of sleep [73], and/or improved daytime alertness [37,38,74,75].

Notwithstanding the prevalence of perinatal depression and compelling rationales for its treatment with bright light, we are aware of only five studies of the effects of bright light therapy on perinatal depression. Three studies were randomized controlled trials and two were open trials. These studies will now be briefly reviewed.

## Studies of the efficacy of bright light therapy for antepartum depression

The general characteristics of studies of bright light treatment for antepartum depression are summarized in Table 1. Results from an open trial by Oren and colleagues [76] showed a significant decrease (by 49%) from baseline in Hamilton Depression Rating Scale (HDRS), Seasonal Affective Disorders Version (SIGH-SAD) after 3 weeks of 10,000 lux bright light therapy. The light was administered for 60 min/day, beginning within 10 min of awakening (*n* = 16). In a subset of participants (*n* = 7) who continued treatment for 5 weeks, mean scores on the SIGH-SAD decreased by 59% from baseline. Compliance was monitored by requiring subjects to call an answering machine daily to report their light use.

**Table 1:** Studies of the efficacy of light therapy for perinatal depression

**Antepartum depression**			
	Oren et al. [76]	Epperson et al. [24]	Wirtz-Justice et al. [77]
Subjects (*n*)	16	10	27
Treatment duration (weeks)	3	5	5
Group randomization	No	Yes	Yes
Time of light sessions	Morning: beginning within 10 min of awakening	Morning: beginning within 10 min of awakening	Morning: beginning within 10 min of awakening
Duration of light sessions (min)	60	60	60
Intensity of light treatment (lux)	10,000	7,000	7,000
Blind ratings	Yes	Yes	Yes
Diagnosis of depression	SIGH-SAD ≥ 20	SIGH-SAD ≥ 20	SIGH-SAD ≥ 20
Main outcome measure(s)	SIGH-SAD	SIGH-SAD	SIGH-SAD, HDRS
**Postpartum depression**			
	Corral et al. 2000 [78]	Corral et al. 2007 [79]	
Subjects (*n*)	2	15	
Treatment duration (weeks)	4	6	
Group randomization	No	Yes	
Time of light sessions	Morning: between 7:00 am and 9:00 am	Morning: between 7:00 am and 9:00 am	
Duration of light sessions (min)	30	60	
Intensity of light treatment (lux)	10,000	10,000	
Blind ratings	No	Yes	
Diagnosis of depression	HDRS =28,29	SIGH-SAD ≥ 15	
Main outcome measure(s)	HDRS	SIGH-SAD	

HDRS = Hamilton Depression Rating Scale (Hamilton, 1967), SIGH–SAD = Structured Interview Guide for the Hamilton Depression Rating Scale, Seasonal Version (Williams, 1988).

Expanding from this initial work, Epperson and colleagues conducted a randomized controlled trial in which participants with antepartum depression were assigned to either 7,000 lux (active, *n* = 5) or 500 lux (placebo, *n* = 5) light [24] for 5 weeks. The treatments were administered for 60 min/day, beginning within 10 min of awakening. To check compliance, subjects called the research clinic daily to log in the time of treatment. SIGH-SAD scores were reduced similarly by bright light (17.3) and the dimmer light (16.6).

Wirz-Justice and colleagues recently conducted a larger randomized controlled trial, in which 27 women with antepartum depression were randomized to 5 weeks of active treatment (7,000 lux bright light, *n* = 16), or placebo (70 lux dim red light, *n* = 11). The treatments were administered for 60 min/day, beginning within 10 min of awakening [77]. Compliance was monitored with participant self-reported daily treatment logs. Participants receiving the bright light treatment showed significantly greater improvement in HDRS with Atypical Depression Supplement (SIGH-ADS) scores than those in the placebo treatment, and categorical analysis revealed that the response rate (HDRS ≥ 50% improvement) at week 5 was significantly greater for bright light (81.3%) than for placebo (45.5%). However, no significant differences between treatments were found in self-reported ratings of depression which included the Montgomery-Asberg Depression Rating Scale (MADRS) and the Beck depression Inventory.

## Studies of the efficacy of bright light therapy for postpartum depression

General characteristics of studies of bright light treatment for postpartum depression are summarized in Table 1. In an open trial by Corral et al. (2000) (*n* = 2), both participants showed substantial clinical improvement (75% reduction in HDRS scores) following 4 weeks of 10,000 lux bright light treatment administered for 30 min between 7:00 am and 9:00 am [78]. However, a randomized controlled trial study by Corral et al., (2007) showed no differences between bright light treatment (10,000 lux, *n* = 10) and placebo (600 lux dim red light, *n* = 5), administered for 6 weeks for 30 min/day between 7:00 am and 9:00 am. Both treatments elicited a 49% reduction in SIGH-SAD scores [79]. Also, similar increases in SIGH-SAD scores were found following withdrawal of the treatments.

In this study, participants were asked to verbally report their treatment compliance weekly to the study physician.

## Conclusions

In summary, antidepressant effects of bright light are well established, and there are several rationales for expecting that bright light might also be efficacious for perinatal depression. In pregnant and/or new mothers, bright light treatment could potentially offset insufficient low levels of light exposure; pathological hormonal profiles; co-morbidities, including disturbed sleep and fatigue; and serotonergic dysregulation which has been linked to inadequate maternal behavior.

However, the efficacy of light therapy for perinatal depression has been reported in only five published studies, which have produced mixed results. These studies have had numerous limitations. First, only three of the five studies used a randomized controlled parallel group design. Second, the sample sizes have been small, perhaps due partly to difficulties in recruiting participants from this population. Third, participant adherence in these studies was only measured via participant self-reports. Adherence may be particularly problematic for this population, who have clear logistical obstacles to treatment. Fourth, studies have not addressed co-morbidities that light could alleviate, including anxiety [80,81], disturbed sleep, and fatigue.

Large randomized controlled trials are needed to determine whether bright light therapy elicits significant improvement in perinatal depression. For many women suffering from antepartum or postpartum depression, bright light is likely preferable to other types of treatment, but further research is needed to address questions about the feasibility of bright light treatment for these women. Comparative effectiveness trials would also fill a gap in existing literature, and may provide an important component of the overall treatment paradigm for women with perinatal depression. More targeted interventions may be feasible, perhaps with a mixed modalities treatment approach, for treating women with depression during pregnancy and/or postpartum. Additionally, further research is needed to determine potential mechanisms of the therapeutic effects of bright light therapy for perinatal depression.

## Competing interests

The authors declare that they have no competing interest

## Authors’ contributions

SC and SY, contributed to the research, writing, and editing of this manuscript. Both authors read and approved the final manuscript.
